# Mechanisms of *Chlorella sorokiniana* and Bacillus combination enhanced tomato (*Solanum lycopersicum*) growth and soil quality

**DOI:** 10.3389/fpls.2026.1766843

**Published:** 2026-04-27

**Authors:** Jinyu Zhao, Liheng Wu, Zhenliang Yu, Shuyan Liu, Wei Ma, Yanru Cui, Weixu Hou, Lina Wang, Xin Tian, Yue Sun, Jinlin Cai

**Affiliations:** 1 Key Laboratory of Marine Resource Chemistry and Food Technology (TUST), Ministry of Education, College of Chemical Engineering and Materials Science, Tianjin University of Science & Technology, Tianjin, China; 2 Heilongjiang Provincial Hydraulic Research Institute, Harbin, China; 3 Jilin Academy of Agricultural Sciences (Northeast Agricultural Research Center of China), Changchun, China

**Keywords:** microbial community structure, microbial fertilizers, soil amendment, soil enzyme activity, tomato

## Abstract

**Introduction:**

Microorganisms are capable of enhancing plant growth by improving the soil microenvironment, supplying nutrients, and facilitating root development. Different microorganisms employ distinct mechanisms to achieve promotion of plant growth.

**Methods:**

This study employed a partitioned plastic greenhouse experiment with three treatments—*Chlorella sorokiniana* (*C. sorokiniana*) inoculation, a Bacillus combination inoculation, and a water control—applied via root irrigation at each tomato growth stage, aiming to investigate the effects of *C. sorokiniana* and Bacillus combination on tomato growth, rhizosphere soil properties, and associated microbial communities.

**Results:**

The results demonstrated that the combination of *C. sorokiniana* and Bacillus significantly promoted plant growth and improved soil quality. *C. sorokiniana* primarily enhanced soil physicochemical properties, as evidenced by a 53.68% decrease in electrical conductivity (EC). It also increased the activities of key soil nitrogen cycle-related enzymes, including urease (42.34%) and protease (24.14%), and elevated soil organic carbon (SOC) content (19.39%). Collectively, these modifications in soil conditions contributed to enhanced plant growth, resulting in increased plant height (11.72%) and stem diameter (9.78%). In contrast, the Bacillus combination enhanced soil nutrient availability (alkaline-hydrolyzable nitrogen (AN) 5.55%, available phosphorus (AP) 21.57%, available potassium (AK) 6.53%) by modulating the structure and function of the soil microbial community. Furthermore, it positively regulated the concentrations of metal ions (Ca^2+^ 0.51%, Mg^2+^ 1.99%), increased stress resistance in tomato plants (peroxidase (POD) 66.18%, superoxide dismutase (SOD) 98.67%, and catalase (CAT) 31.79%), and consequently enhanced plant growth metrics, including height (17.36%) and stem diameter (18.36%). These changes in soil physicochemical parameters may be related to increased rhizosphere microbial activity. Further correlation analysis indicated a positive relationship between the relative abundance of the Patescibacteria phylum and soil EC values.

**Discussion:**

This study elucidates key mechanisms underlying growth promotion in tomatoes and establishes a theoretical foundation for the application of Bacillus combination and *C. sorokiniana* as microbial fertilizers.

## Graphical Abstract

**Figure:**
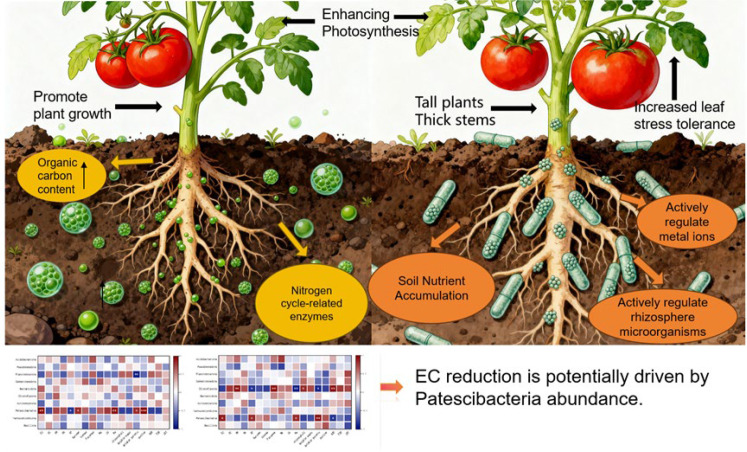

## 1 Introduction

According to data from the Food and Agriculture Organization of the United Nations, tomato (*Solanum lycopersicum* L.) is a globally important vegetable crop, with an annual cultivated area of approximately 5.12 million hectares and a yearly production of about 188.5 million tons (Food and Agriculture Organization of the United Nations, 2024). Tomatoes are utilized both fresh and in processed forms due to their content of essential nutrients and bioactive compounds, which are associated with notable health benefits. However, tomato cultivation suffers from significant challenges, including environmental pollution, a decline in beneficial soil microorganisms, and soil acidification (Cruz-López et al., 2024; Fu et al., 2017; Lyu et al., 2020; Chanthini et al., 2025). A common practice to enhance crop yield has been the excessive and indiscriminate application of chemical fertilizers over prolonged periods (Harman et al., 2020; Saleem et al., 2024). The residual presence of toxic chemicals from fertilizers and pesticides adversely affects the natural environment, disrupts soil ecosystems, and poses risks to human health. In contrast, microbial fertilizers represent an environmentally sustainable and effective alternative, demonstrating significant potential in promoting crop growth and enhancing soil quality.

Microbial fertilizers enhance plant growth and nutrient uptake via synergistic interactions within the rhizosphere (Al-Tammar and Khalifa, 2022). Owing to their demonstrated efficacy in increasing crop yield and quality, these fertilizers have emerged as a widely recognized, environmentally sustainable alternative to conventional chemical inputs (Taheri et al., 2025; Calvo et al., 2014). The use of microalgae as biofertilizers in modern agriculture has been widely investigated. Microalgae can enhance soil physicochemical properties through mechanisms including nitrogen fixation, carbon sequestration, and growth-hormone secretion. These activities concurrently stimulate the proliferation of beneficial soil microorganisms. These effects collectively enhance plant growth and nutrient uptake (Renuka et al., 2018, 2016). Algal-based products improve crop productivity and can be utilized as multifunctional agricultural inputs, including biofertilizers, liming materials, soil amendments, plant bio-stimulants, and blended fertilizer formulations (Choulot et al., 2022). In contrast to microbial inoculants, algae are rich in diverse nutrients including fiber, minerals, proteins, vitamins, and fatty acids, which contribute to plant nutrition (Kumar et al., 2021). Furthermore, microalgal extracts contain abundant natural plant growth regulators (PGRs) and beneficial trace minerals, including but not limited to coenzymes, cytokinins, and gibberellins. These bioactive compounds effectively modulate root system architecture development, thereby enhancing crop performance through improved nutrient acquisition and stress resilience (Hakim and Patel, 2020). Microalgae fertilizers significantly enhance root development by increasing root length and biomass, consequently improving crop stress tolerance to environmental adversities. Moreover, microalgal fertilizers exhibit pronounced bio-stimulant effects, significantly enhancing seed germination rates, stimulating shoot growth, increasing chlorophyll content, and improving photosynthetic efficiency (Augier and Santimone, 1978).

Among the functional microorganisms utilized in Microbial fertilizers, strains of the genus *Bacillus* serve as prominent representatives, with well-documented growth-promoting benefits (Flaviano et al., 2025). Research indicates that *Bacillus cereus* promotes plant growth through multiple mechanisms, including the synthesis of indole-3-acetic acid (IAA), production of 1-aminocyclopropane-1-carboxylate (ACC) deaminase, and solubilization of phosphate (Kulkova et al., 2023). It also enhances plant biomass accumulation, the content of essential nutrients, and bioactive compounds (such as antioxidant enzymes and total soluble sugars), supporting the growth of crops including soybean, maize, rice, and wheat. Certain strains of *Bacillus cereus* can promote plant growth under abiotic stresses such as drought, salinity, and heavy metal contamination. Additionally, they indirectly stimulate plant growth by producing extracellular enzymes, synthesizing antibiotic lipopeptides, or triggering induced systemic resistance (ISR) (Kulkova et al., 2023). Pot experiments have demonstrated that applying *Bacillus subtilis* and *Bacillus polymyxa* as biofertilizers significantly enhanced tomato root length, root surface area, root volume, and root activity (Luo et al., 2025).

Current research has extensively documented the plant growth-promoting (PGP) effects of *Bacillus* and *C. sorokiniana*, with particular emphasis on their symbiotic mechanisms. However, the mechanistic divergences between these two microorganisms in plant growth promotion remain poorly characterized, representing a critical knowledge gap that warrants in-depth investigation. The primary objective of this study is to elucidate the intrinsic mechanisms underlying the differential plant growth-promoting effects between *C. sorokiniana* and a Bacillus combination, with particular focus on their distinct regulation of soil properties and rhizosphere microecology. However, current studies have predominantly centered on *Chlorella vulgaris*, leaving the agricultural potential of the more rapidly growing and environmentally resilient *C. sorokiniana* largely underexplored. Two major research gaps persist: first, systematic validation of the specific growth-promoting effects of *C. sorokiniana* on crops such as tomato is still lacking; second, the physiological and molecular mechanisms underlying the interactions between this microalga and crops remain poorly understood. Specifically, this investigation will: (1) Systematically evaluate the differential effects of *C. sorokiniana* and the Bacillus combination on tomato growth parameters (stem diameter, plant height) and leaf physiological indices (SPAD value, net photosynthetic rate); (2) Quantitatively assess their distinct impacts on rhizosphere soil characteristics, including pH dynamics, organic matter content, and nutrient availability (alkali-hydrolyzable nitrogen, available phosphorus, available potassium); (3) Mechanistically elucidate their species-specific modulation of soil microbial community structure (via 16S rRNA/ITS sequencing) and explore potential feedback mechanisms between microbial changes and electrical conductivity.

## 2 Materials and methods

### 2.1 Microbial fertilizers

Bacillus combination and *C. sorokiniana* were maintained in our laboratory. The Bacillus consortium consisted primarily of *Bacillus subtilis* WLH-1, *Bacillus cereus* WLH-2 and Halophiles SY-1. Halophiles SY-1, independently isolated in our laboratory, exhibits high salt tolerance (up to 8% NaCl) and a strong capacity for phosphorus solubilization. Furthermore, no significant antagonism was observed when it was co-cultured with the two Bacillus strains. These characteristics suggest that strain SY-1 has the potential to act synergistically with Bacillus species to promote plant growth. To prepare the composite inoculum, Bacillus strains WLH-1 and WLH-2 were cultured in LB medium (10 g peptone, 5 g yeast extract, 10 g NaCl per liter), while strain SY-1 was cultured in 2216E medium (5 g peptone, 1 g yeast extract, 0.01 g ferric phosphate, 1 L aged seawater, pH 7.5–7.6). After being cultured to the logarithmic growth phase, cells from each strain were harvested by centrifugation, washed, and resuspended in sterile physiological saline. These suspensions were first mixed in equal volumes. Based on the determined effective viable counts—2.3 × 10¹¹ CFU/mL for WLH-1, 1.9 × 10^10^ CFU/mL for WLH-2, and 2.6 × 10¹¹ CFU/mL for strain SY-1—the suspensions were further diluted and mixed at a final volumetric ratio of 4:4:1 (WLH-1: WLH-2: SY-1). The final composite inoculum had an effective viable cell concentration of 1.4 × 10¹¹ CFU/mL. The concentration of *C. sorokiniana* was 1.4 × 10^11^cfu/mL.

### 2.2 Experimental site, design, and sampling

This experiment was conducted in Harbin City, Heilongjiang Province from June to October 2024. The region features a mid-temperate continental monsoon climate, with an annual average temperature of 5.4 °C, average atmospheric pressure of 997 hPa, and average wind speed of 2.4 m/s. Annual precipitation averages approximately 501.5 mm, primarily concentrated between July and September, while the annual average relative humidity is 62.7%.

This experiment was conducted in soil plots located within a plastic greenhouse, with three treatment groups established: the *C. sorokiniana* treatment group, the Bacillus combination treatment group, and the control group (CK). Each treatment was replicated three times, resulting in a total of 9 experimental plots. The individual plot size was 1 m × 6 m (area of 6 m²). A completely randomized block design was employed in a greenhouse environment to minimize the potential impact of environmental heterogeneity on the experimental results. To prevent cross-contamination of water and microorganisms between different treatments, a 1 m wide buffer zone was established between the plots. For each treatment group, 1 L of bacterial solution per mu was diluted at a 1:9 volume ratio and uniformly sprayed onto the soil around the crop roots. The CK group received an equal volume of clean water. All treatments were applied every 15 days, with a total of two applications, maintaining an interval of at least 2–3 days from pesticide or herbicide applications. Planting density is set at 40 cm × 40 cm spacing between plants and rows. Water management employed subsurface drip irrigation throughout the growth cycle, with precision irrigation based on volumetric soil moisture monitored by sensors: thorough irrigation at planting (8–10 m³/667 m²), followed by establishment irrigation 3–5 days later (5–7 m³/667 m²). Water was moderately restricted from flowering to initial fruit set, maintaining soil moisture at 50%–60% of field capacity. Irrigate during early, peak, and late fruiting stages when soil moisture falls below 60–70%, 70–80%, and 70–80% of field capacity, respectively, with single irrigation volumes of 6–8 m³/667 m², 8–10 m³/667 m², and 6–8 m³/667 m². All treatments underwent conventional cultivation practices including suckering, vine training, and topping to maintain uniform canopy structure. At the conclusion of the trial, soil and plant samples were collected. Soil samples were obtained using the five-point sampling method, with a total volume of 1 kg collected per sampling event. Plant samples comprised 3–5 healthy functional leaves (approximately 10 g) for subsequent indicator measurements and analysis.

Rhizosphere soil was collected using the root-shaking method after gently uprooting tomato plants. After sieving (2mm), samples were portioned into 200g units, labeled, and transported back to the laboratory in ice or dry ice. Some samples were air-dried for nutrient and enzyme activity analysis, while the remainder were stored at -80 °C for microbial analysis. Leaf samples were collected at midday under full sunlight, maintaining consistency and growth vigor. Wrapped in aluminum foil, they were prioritized for rapid freezing in liquid nitrogen and stored at -80 °C. If liquid nitrogen was unavailable, dry ice or ice packs were used for transport to ensure sample integrity and viability. The above-ground height of tomato plants (i.e., the vertical distance between the highest natural growth point and the soil surface) and stem diameter were measured every 9 days for a total of 4 measurements to evaluate seedling growth status.

### 2.3 Basic soil properties

The methods for analyzing the soil samples collected at different time periods are as follows. Soil pH was measured with a pH meter, and soil electrical conductivity (EC) was determined with a conductivity meter (Maeda et al., 2023). Soil organic carbon (SOC) was quantified using the potassium dichromate oxidation method (Ivezić et al., 2016). Available phosphorus (AP) and available nitrogen (AN) were determined by the sodium bicarbonate-molybdenomolybdate spectrophotometric method and the alkali-hydrolyzation diffusion method, respectively (Holford, 1997). The contents of available potassium (AK), sodium (Na^+^), calcium (Ca^2+^), and magnesium (Mg^2+^) in the soil were measured using the Ammonium acetate extraction inductively coupled plasma optical emission spectrometry (AA-ICP-OES) method (Yuan et al., 2025; Zhao et al., 2018).

### 2.4 Soil enzyme activity

The methods for determining soil enzyme activities are as follows. Soil urease activity was measured using the sodium phenol colorimetric method (Guo et al., 2012). Soil invertase activity was determined by the 3, 5-dinitrosalicylic acid (DNS) method (Wang et al., 2020). Soil protease activity was assessed via the casein-ninhydrin colorimetric method (Zhang et al., 2023). Soil catalase (CAT) activity was measured by potassium permanganate titration (Zheng et al., 2017).

### 2.5 Basic characteristics and resistance capacity of leaves

The following biochemical parameters were determined using their respective methods: SOD activity by the nitroblue tetrazolium method (Beauchamp and Fridovich, 1971), POD activity by the guaiacol method (Maehly and Chance, 1954), catalase (CAT) activity by the ultraviolet absorption method (Xiong et al., 2018; Aebi, 1984), MDA content by the thiobarbituric acid method, soluble sugar content by the anthrone colorimetric method (Huang et al., 2025), proline content by the acid-ninhydrin colorimetric method, soluble protein content by the Coomassie Brilliant Blue method (Bradford, 1976), and chlorophyll content by anhydrous ethanol extraction.

### 2.6 DNA extractions and illumina sequencing

Total genomic DNA was extracted from soil samples using the E.Z.N.A.™ Mag-Bind Soil DNA Kit (OMEGA, Cat. No. M5635-02) following the manufacturer’s protocol. DNA concentration was measured using a Qubit 4.0 Fluorometer (Thermo Fisher Scientific, USA).

The V3–V4 hypervariable region of the bacterial 16S rRNA gene was amplified using primers 341F (CCTACGGGNGGCWGCAG) and 805R (GACTACHVGGGTATCTAATCC). Each 30 µL PCR reaction contained 2 µL of DNA template (10 ng/µL), 1 µL of each primer (10 µM), and 15 µL of 2× Hieff^®^ Robust PCR Master Mix (Yeasen, 10105ES03, China). Thermal cycling was performed on an Applied Biosystems 9700 instrument as follows: initial denaturation at 95 °C for 3 min; 5 cycles of 95 °C for 30 s, 45 °C for 30 s, and 72 °C for 30 s; followed by 20 cycles of 95 °C for 30 s, 55 °C for 30 s, and 72 °C for 30 s; with a final extension at 72 °C for 5 min. PCR products were verified by electrophoresis on a 2% agarose gel.

Amplicons were purified using Hieff NGS™ DNA Selection Beads. Libraries were constructed by Sangon Biotech (Shanghai) Co., Ltd. using Illumina adapters and indices, quantified with a Qubit Fluorometer, and pooled in equimolar ratios. Sequencing was performed on an Illumina MiSeq platform (Illumina, USA). The raw reads were bioinformatically processed to calculate α-diversity indices, determine taxonomic classification, and perform redundancy analysis (RDA).

### 2.7 Data analysis

Data processing and statistical analyses were performed using Microsoft Excel 2019, IBM SPSS Statistics 21, and Origin 2021. The significance of differences among treatments was assessed by one-way ANOVA, with results indicated by distinct lowercase letters (p < 0.05). Redundancy Analysis (RDA) and Pearson correlation analysis were conducted using Origin to identify key environmental factors driving microbial community dynamics and to visualize their correlations through heatmaps, respectively.

## 3 Results

### 3.1 Effect of *C. sorokiniana* and Bacillus combination on growth of tomato

**Figure 1:**
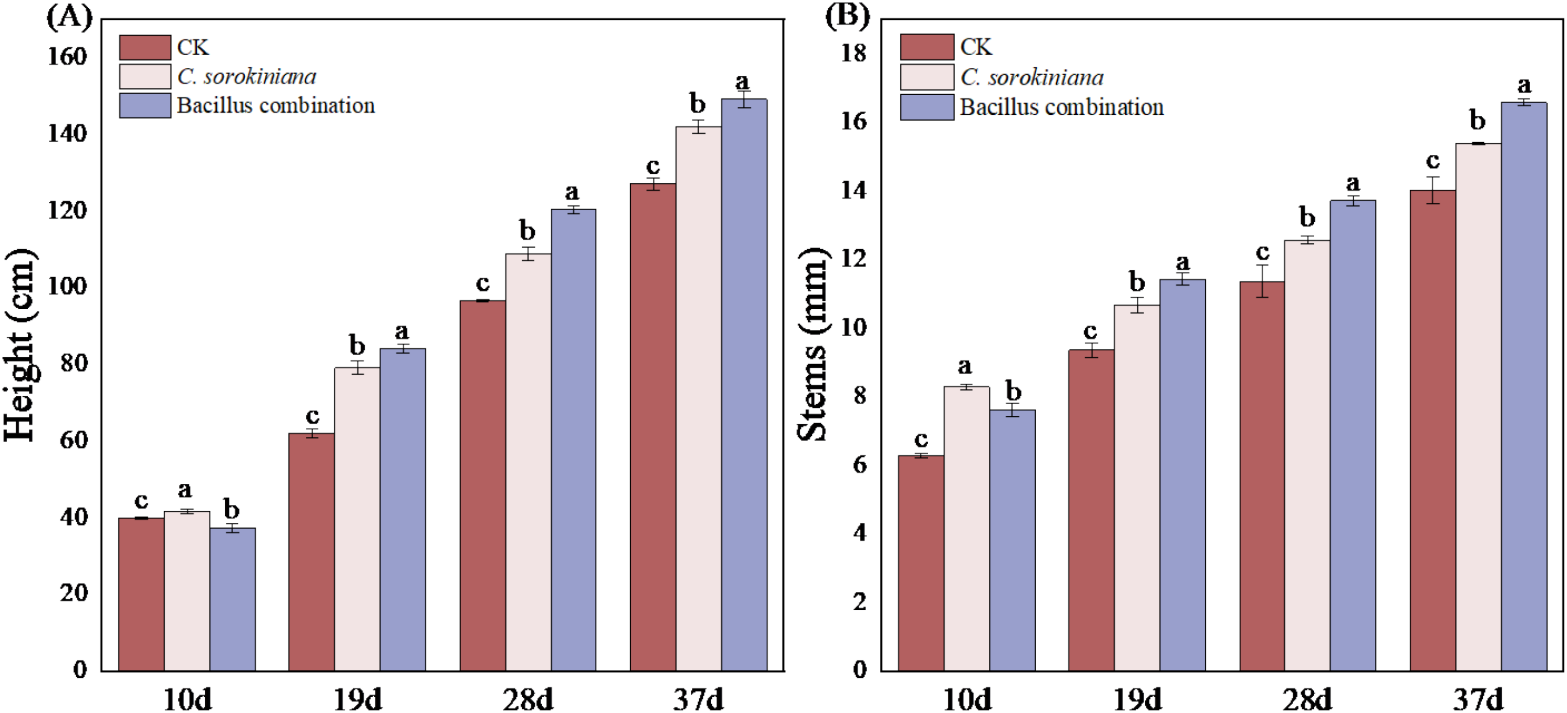
Effects of microbial strain addition on tomato plant phenotypes. **(A)** Height of tomato plants aboveground; **(B)** Stem length of tomato plants.

As illustrated in **Figure 1**, tomato growth was significantly improved by inoculation of *C. sorokiniana* and Bacillus combination. Both plant height (**Figure 1A**) and stem diameter (**Figure 1B**) were significantly increased compared with CK. Although the growth-promoting effect of *C. sorokiniana* was stronger than that of the Bacillus combination in the early growth stage, the efficacy of the *Bacillus* treatments increased progressively over time. Eventually, they surpassed *C. sorokiniana* and maintained this superior promoting effect until the final harvest.

### 3.2 Effect of *C. sorokiniana* and Bacillus combination on tomato leaves

**Figure 2:**
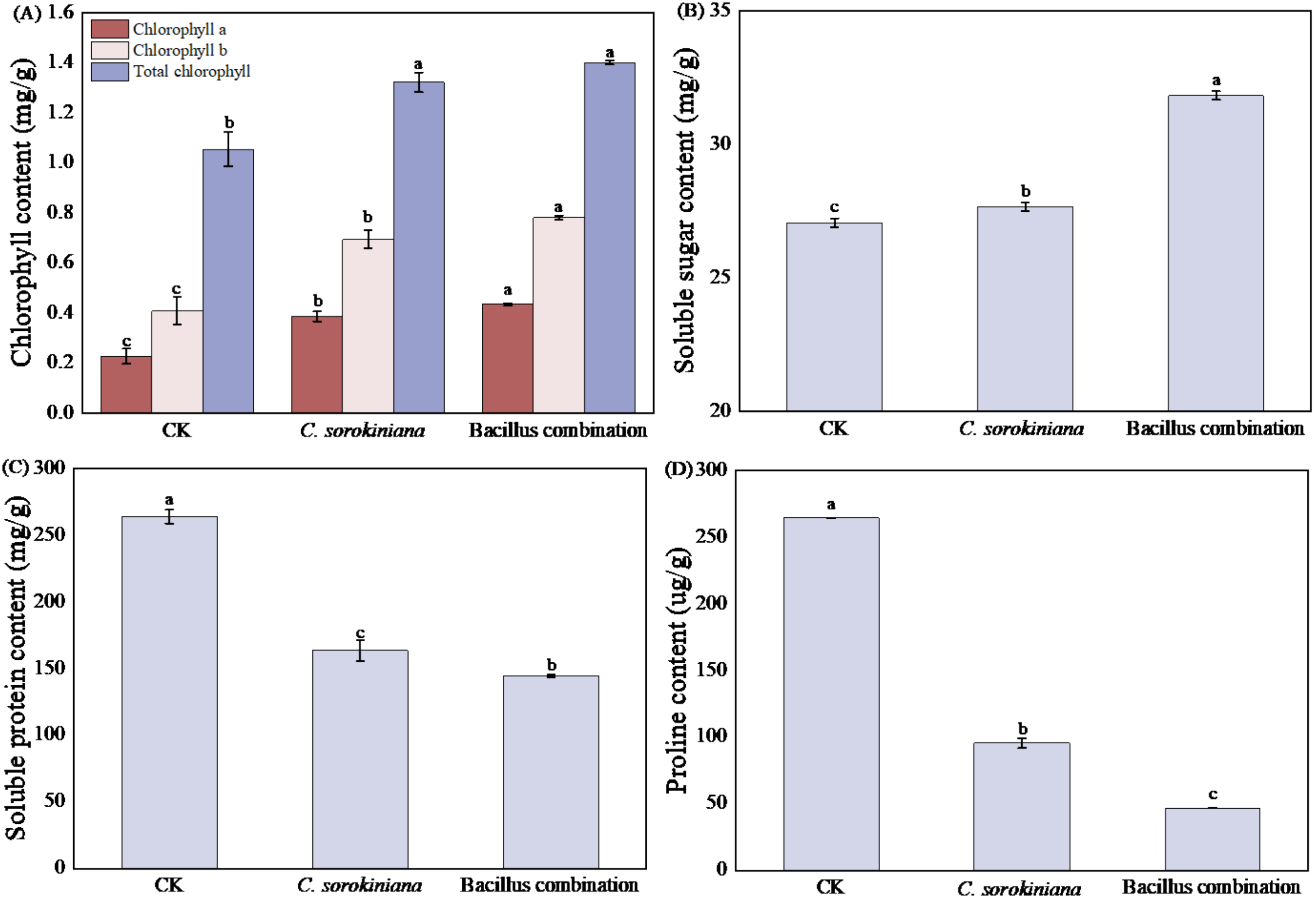
Effect of strain addition on physiological and biochemical indexes of leaves. **(A)** Chlorophyll content; **(B)** Soluble sugar content; **(C)** Soluble protein content; **(D)** Proline content.

Leaves are the primary site of photosynthesis in plants. Chlorophyll content, soluble sugar content, soluble protein, and proline were measured and compared in tomato leaves subjected to different treatments. Results shown in **Figure 2A** indicate that compared to the CK, chlorophyll a, chlorophyll b, and total chlorophyll content in tomato leaves significantly increased after treatment with *C. sorokiniana* and Bacillus combination. Both treatments resulted in significantly higher soluble sugar content than CK (**Figure 2B**). However, Soluble protein (**Figure 2C**) and proline (**Figure 2D**) content in tomato leaves treated with *C. sorokiniana* and the Bacillus combination were significantly lower than CK.

**Figure 3:**
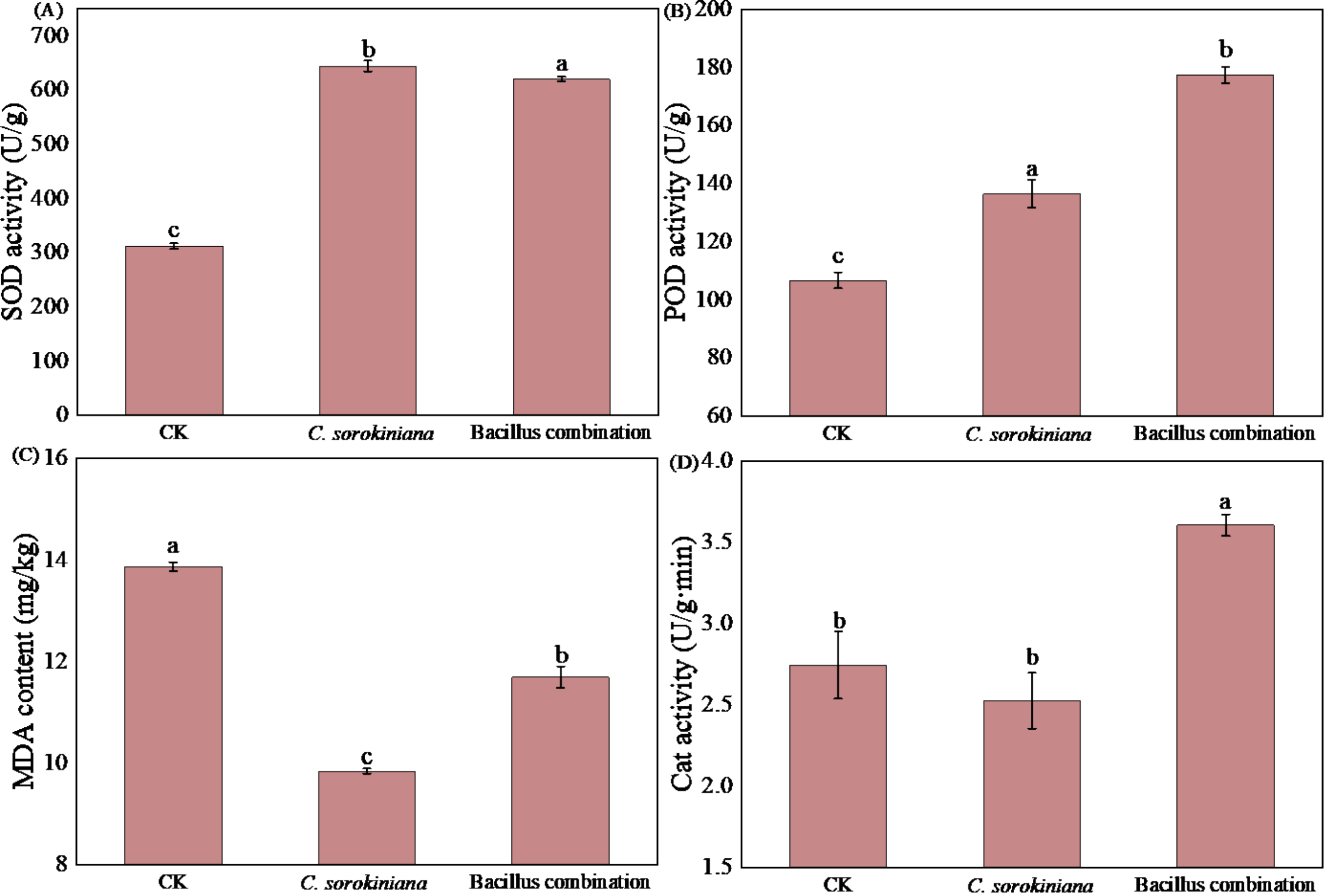
Effect of strain addition on leaf resistance capacity. **(A)** SOD activity; **(B)** POD activity; **(C)** MDA content; **(D)** Cat activity.

As shown in **Figure 3**, the inoculation of Bacillus combination significantly enhanced antioxidant enzyme activity in tomato leaves, with increases of 66.18%, 98.67%, and 31.79% in POD, SOD, and CAT activities compared to the CK. Treatment with *C. sorokiniana* significantly increased POD and SOD activities by 28% and 106%, respectively. Although CAT activity decreased, the difference compared to CK was not significant. MDA content was lower in both treatments than CK, with *C. sorokiniana* treatment reducing by 17.28% and Bacillus combination treatment by 10.98%.

### 3.3 Effects of *C. sorokiniana* and Bacillus combination on soil properties

Compared with CK, treatment with *C. sorokiniana* resulted in a decrease in soil pH, whereas the *Bacillus* combination treatment led to an increase. (**Table 1**). Both treatments consistently reduced the soil EC, with decreases of 53.68% and 28.09% for the *C. sorokiniana* and Bacillus combination treatments, respectively.

**Table 1:** Effect of strain addition on soil pH and electrical conductivity (EC).

Treatment	pH	EC(μs·cm^−1^)
CK	8.26 ± 0.02^b^	559 ± 11.6^a^
*C. sorokiniana*	8.18 ± 0.05^c^	259 ± 3.33^c^
Bacillus combination	8.36 ± 0.01^a^	402 ± 5.36^b^

**Figure 4:**
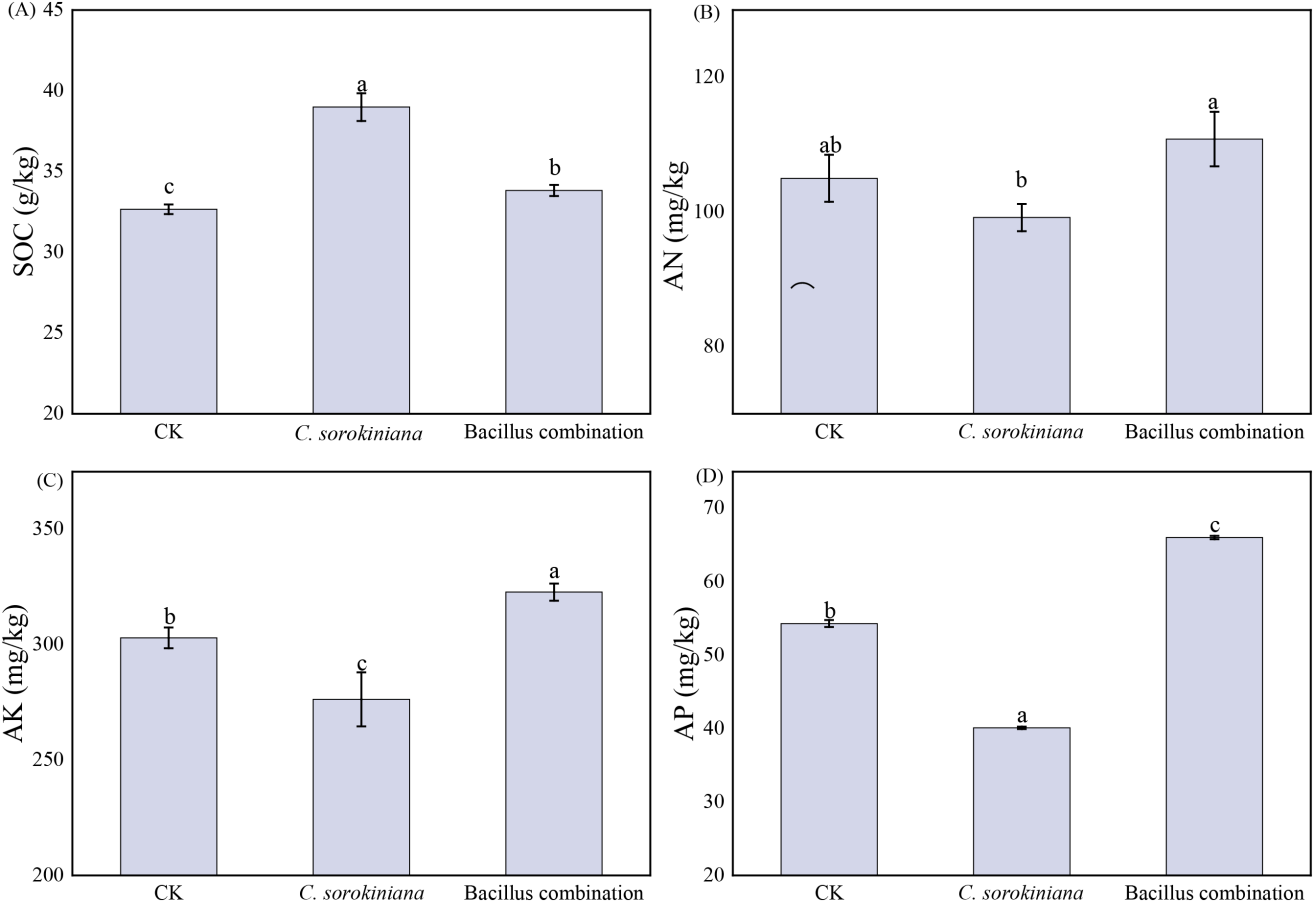
Effect of strain addition on soil nutrient concentrations. **(A)** SOC; **(B)** AN; **(C)** AK; **(D)** AP.

The inoculation of microbial inoculants affects the decomposition rate of organic matter in soil. SOC content increased under both treatments relative to the CK (**Figure 4A**). *C. sorokiniana* treatment increased by 19.39%, while the Bacillus combination treatment increased by 3.58%. Following both treatments, soil nutrient content exhibited fluctuating changes. The inoculation of Bacillus combination significantly increased the contents of AN (**Figure 4B**), AK (**Figure 4C**), and AP (**Figure 4D**) by 5.55%, 6.53% and 21.57%, respectively, compared to the CK. Conversely, the inoculation of *C. sorokiniana* resulted in decreased levels of AN, AK, and AP, with significant reductions of 5.55%, 8.81% and 26.15%, compared to the CK, respectively.

### 3.4 Effects of *C. sorokiniana* and Bacillus combination on soil enzyme activity

**Figure 5:**
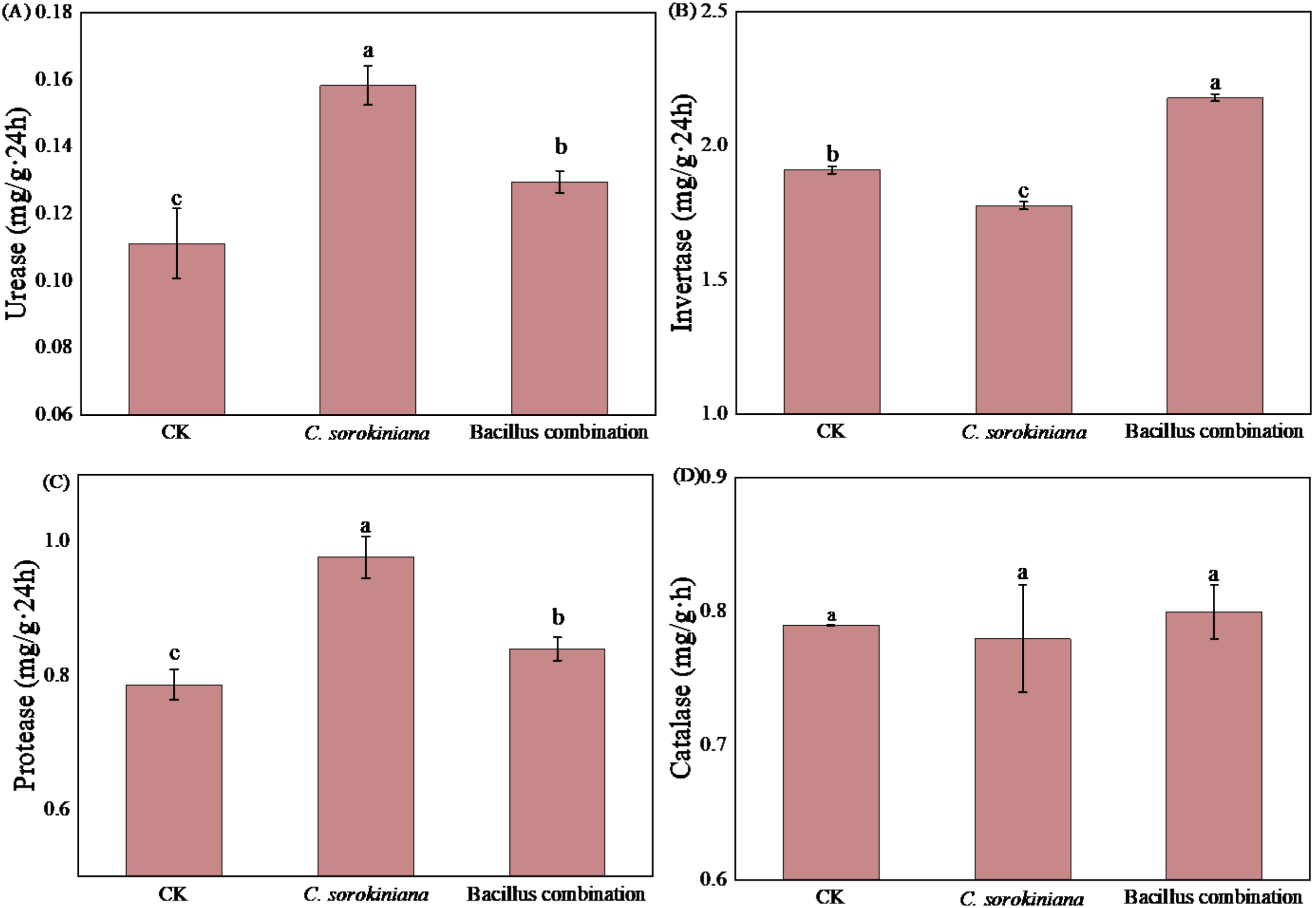
Effect of strain addition on soil enzyme activity. **(A)** Urease; **(B)** Invertase; **(C)** Proteases; **(D)** Catalase.

The effects of two treatments on soil enzyme activities are shown in **Figure 5** Following application of *C. sorokiniana*, the activities of urease and protease significantly increased, while Invertase activity significantly decreased. After inoculation of the Bacillus combination, the activities of Invertase, urease, and protease all significantly increased. For urease, *C. sorokiniana* treatment increased activity by 42.34%, while the Bacillus combination increased activity by 17.12% (**Figure 5A**). For Invertase, the Bacillus combination treatment increased activity by 14.14% (**Figure 5B**). For protease, *C. sorokiniana* treatment increased activity by 24.14%, while the Bacillus combination treatment increased activity by 6.73% (**Figure 5C**). Neither treatment significantly affected catalase activity (**Figure 5D**).

### 3.5 Effects of *C. sorokiniana* and Bacillus combination on soil metal elements

**Figure 6:**
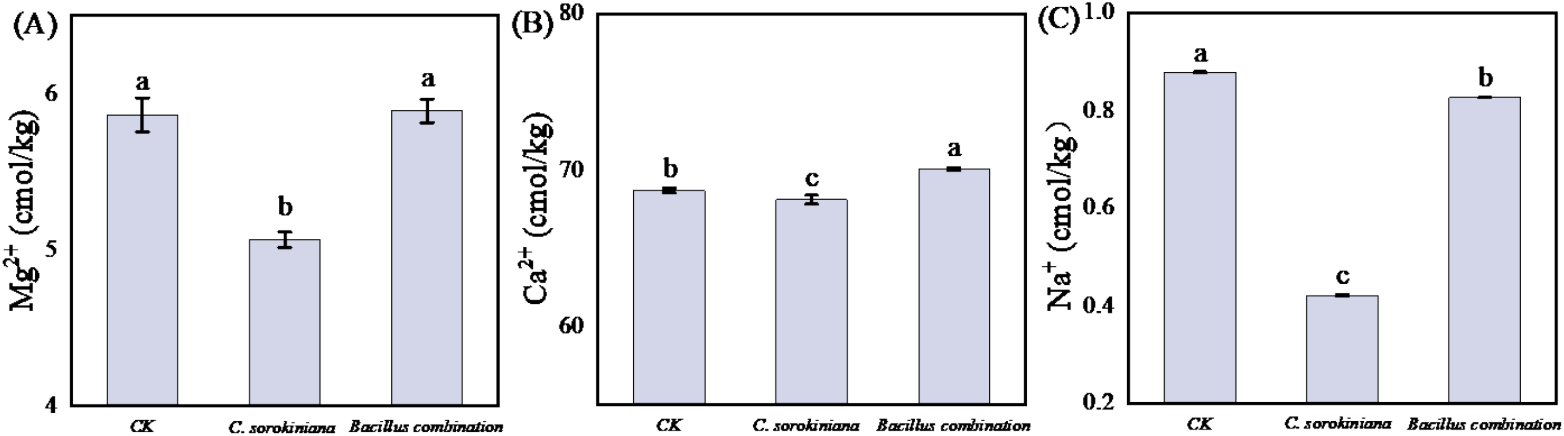
Effect of strain addition on soil metal elements content. **(A)** Mg^2+^; **(B)** Ca^2+^; **(C)** Na^+^.

The contents of three metallic elements (Mg^2+^, Ca^2+^, and Na^+^) in soils from different treatment groups were determined, and the results are shown in **Figure 6**. Compared with the CK, the treatment with *C. sorokiniana* led to a decrease in Mg^2+^ (**Figure 6A**) and Ca^2+^ (**Figure 6B**) contents in the soil. In contrast, the treatment with Bacillus combination resulted in an increase in both Mg^2+^ and Ca^2+^ contents. Treatment with *C. sorokiniana* decreased soil Mg^2+^ and Ca^2+^ content by 13.65% and 0.86%, respectively. In contrast, the Bacillus combination increased these ions by 0.51% (Mg^2+^) and 1.99% (Ca^2+^). Regarding Na^+^ (**Figure 6C**), its soil content exhibited a decreasing trend regardless of treatment with either *C. sorokiniana* or the Bacillus combination, with reductions of 52.03% and 5.86%, respectively.

### 3.6 Effects of *C. sorokiniana* and Bacillus combination on soil microbial diversity and community function

**Figure 7:**
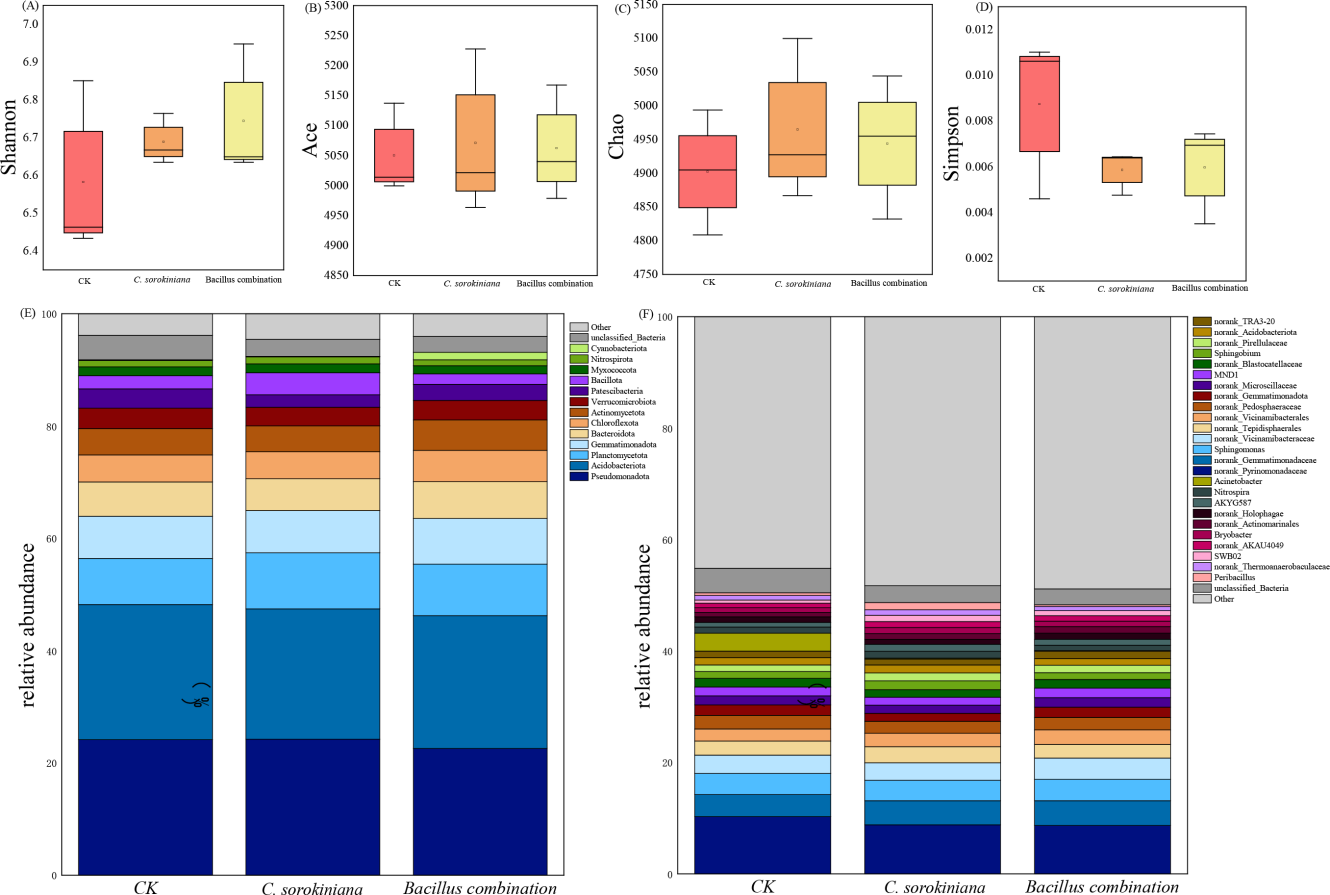
Microbial community diversity and composition. **(A)** Shanno; **(B)** Chao; **(C)** Ace; **(D)** Simpson; **(E)** Phylum-level relative abundance; **(F)** Genus-level relative abundance.

The alpha diversity indices of bacterial communities in soils subjected to different treatments are shown in the **Figure 7**. The Shannon index value was highest in soil inoculated with the Bacillus combination (**Figure 7A**). Meanwhile, soil inoculated with *C. sorokiniana* exhibited the highest Chao (**Figure 7B**) and Ace indices (**Figure 7C**), along with the lowest Simpson index (**Figure 7D**).

At the phylum level, soil microbial communities inoculated with *C. sorokiniana* and the Bacillus combination exhibited consistent phylum composition but differing abundance levels. As shown in **Figure 7E**, twelve dominant bacterial phyla (average relative abundance >1%) were commonly present across all three samples at the phylum level. These included Pseudomonadota, Acidobacteriota, Planctomycetota, Gemmatimonadota, Bacteroidota, Chloroflexota, Actinomycetota, Verrucomicrobiota, Patescibacteria, Bacillota, Myxococcota, Nitrospirota.Additionally, the Bacillus combination treatment group contained the dominant bacterial phylum Cyanobacteriota. Among the 12 dominant bacterial phyla, the *C. sorokiniana* treatment group increased the relative abundance of four phyla: Pseudomonadota, Planctomycetota, Bacillota, and Nitrospirota. The Bacillus combination treatment group increased the relative abundance of six phyla: Planctomycetota, Gemmatimonadota, Bacteroidota, Chloroflexota, Actinomycetota, and Cyanobacteriota.

Soil microbial communities also exhibited differences in abundance at the genus level following inoculation with *C. sorokiniana* and Bacillus combination (**Figure 7F**). Community composition analysis revealed that approximately 40% of high-quality sequenced reads could not be identified at the bacterial genus level, indicating a substantial proportion of microorganisms remained unclassified within the current taxonomic framework. Among the successfully classified sequences, *Sphingomonas* showed the highest relative abundance, followed by the *Sphingobium*. Differential relative abundance of specific genera was observed between treatments: *Sphingomonas* represented 4.50% in the *C. sorokiniana* treatment, declining to 3.94% in the Bacillus combination treatment, while *Sphingobium* accounted for 1.81% in the *C. sorokiniana* treatment and 1.20% in the Bacillus combination treatment.

**Figure 8:**
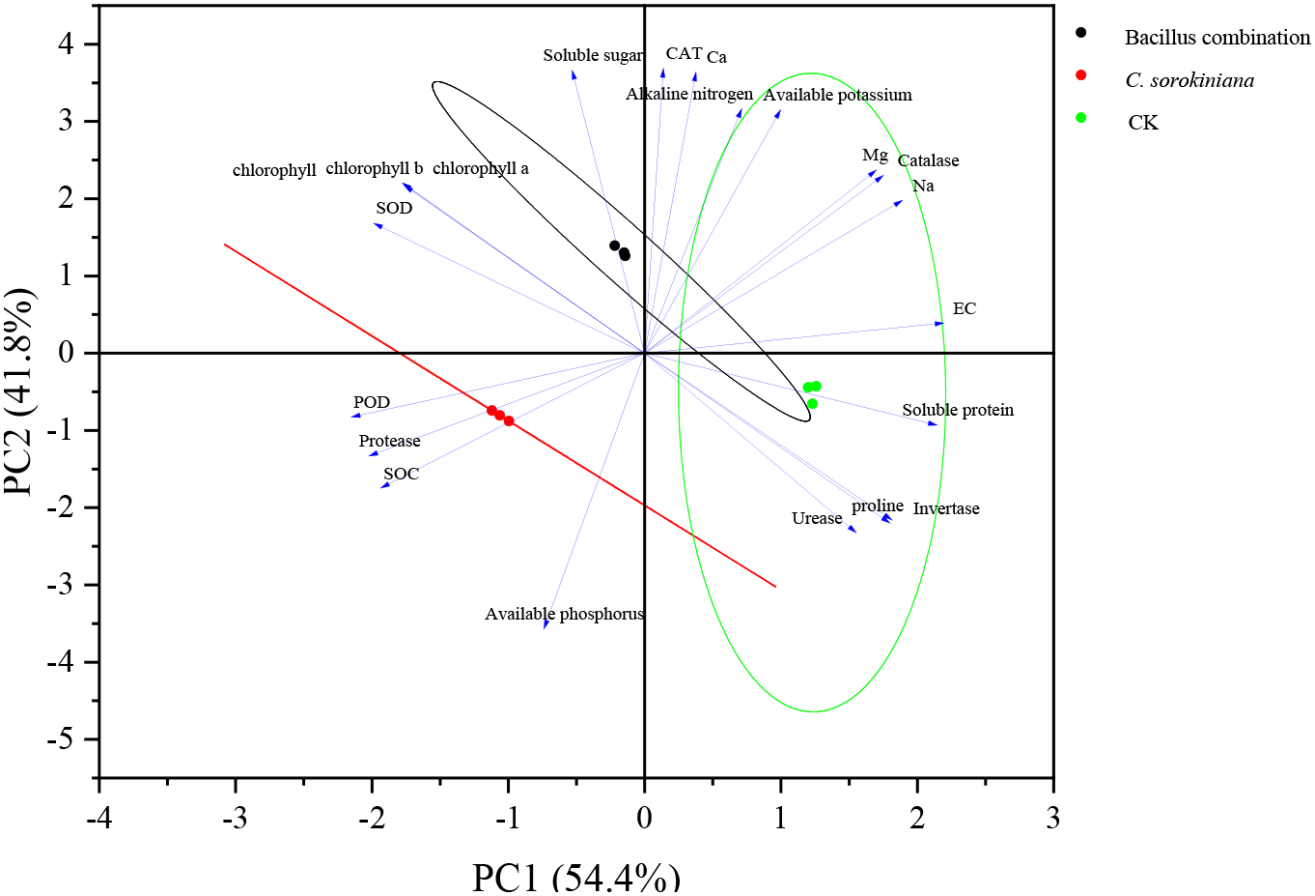
RDA of bacterial community composition and soil environmental factors.

RDA analysis revealed correlations between various quantitative environmental factors and the structure of rhizosphere bacterial communities. As shown in **Figure 8**, SOC, protease activity, and POD activity were the primary environmental factors influencing the formation of *C. sorokiniana* rhizosphere bacterial communities, while exhibiting negative correlations with EC, Ca^2+^, Mg^2+^, and Na^+^. In contrast, soluble sugar, CAT, Ca^2+^, alkaline nitrogen, and available potassium were identified as the primary environmental factors influencing the formation of the Bacillus combination rhizosphere bacterial community.

**Figure 9:**
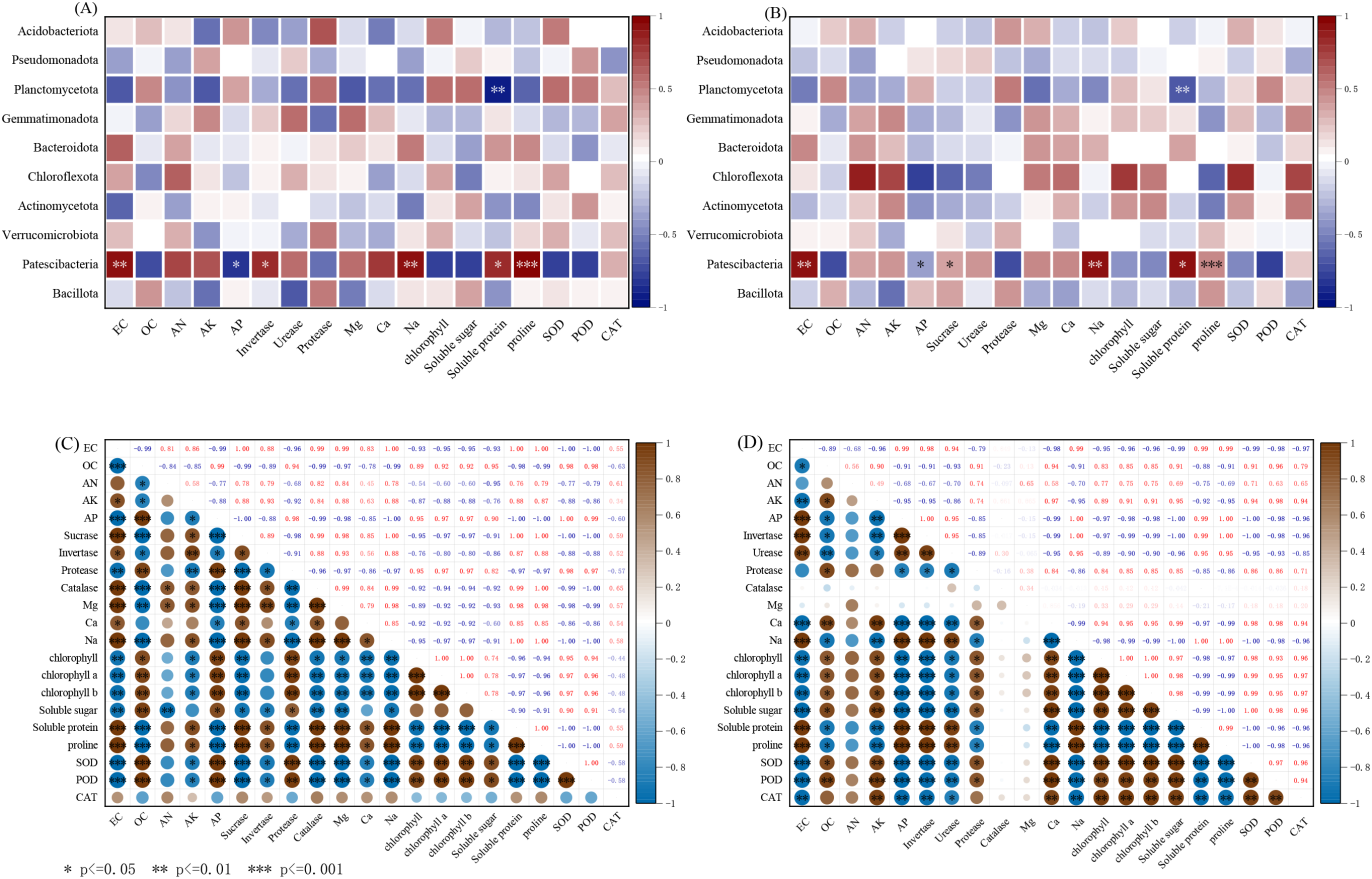
Correlations between soil environmental factors and bacterial communities under different treatments. **(A)** The Spearman correlations between the soil environmental variables and the relative abundance of bacterial communities at the phylum levels. (*C. sorokiniana*); **(B)** The Spearman correlations between the soil environmental variables and the relative abundance of bacterial communities at the phylum levels (Bacillus combination); **(C)** Pearson correlation among environmental variables (*C. sorokiniana*); **(D)** Pearson correlation among environmental variables (Bacillus combination).

Spearman correlation results between inoculated *C. sorokiniana* sample indicators are shown in **Figure 9A**. The proportions of microorganisms such as the *Patescibacteria* phylum and *Planctomycetota* phylum in soils inoculated with *C. sorokiniana* showed significant correlations with environmental factors. *Patescibacteria* showed significant positive correlations with EC, invertase activity, Na^+,^soluble protein, and proline, while exhibiting a significant negative correlation with AP. *Planctomycetota* demonstrated a significant negative correlation with soluble protein. Pearson correlation results among sample indicators are shown in **Figure 9B**. EC exhibited significant positive correlations with AK, Ca^2+^, Mg^2+^, Na^+^, invertase, urease, soluble protein, and proline, while showing significant negative correlations with OC, AP, chlorophyll, chlorophyll a, chlorophyll b, SOD, and POD. OC showed significant positive correlations with AP, chlorophyll, chlorophyll a, chlorophyll b, soluble sugars, SOD, and POD, and significant positive correlations with AN, AK, invertase, urease, soluble protein, and proline; AK showed a significant positive correlation with invertase and urease, while AP showed a significant positive correlation with chlorophyll a, chlorophyll b, and soluble sugars. Ca^2+^, Mg^2+^, and Na showed a significant positive correlation with soluble proteins and proline, and a significant negative correlation with chlorophyll, chlorophyll a, chlorophyll b, and soluble sugars. Chlorophyll, chlorophyll a, chlorophyll b, and soluble sugars showed significant positive correlations with SOD and POD; they showed significant negative correlations with soluble proteins and proline. Soluble proteins and proline showed significant negative correlations with SOD and POD.

The Spearman correlation results between Bacillus combination samples and indicators are shown in **Figure 9C**. The proportions of microorganisms such as Patescibacteria and Chloroflexota in soils inoculated with the Bacillus combination showed significant correlations with environmental factors. Patescibacteria showed significant positive correlations with EC, AP, Na^+^, and soluble protein, and a significant negative correlation with SOD. Chloroflexota exhibited significant positive correlations with AN, protease, chlorophyll, and SOD, and significant negative correlations with AP, Na^+^, and soluble protein. Pearson correlation results among sample indicators are shown in **Figure 9D**. Unlike inoculation with *C. sorokiniana*, EC showed a significant positive correlation with AP and a significant negative correlation with AK and Ca^2+^; OC showed a significant positive correlation with AK and Ca^2+^ and a significant negative correlation with AP. Indicators showing significant correlations with AK and AP exhibited opposite trends compared to inoculation with *C. sorokiniana*. Notably, the significant correlations of all indicators with Ca^2+^ were opposite to those observed with *C. sorokiniana* inoculation.

## 4 Discussion

### 4.1 Effects of *C. sorokiniana* and Bacillus combination on tomato plant growth, basic leaf characteristics, and stress resistance

In this study, we found that during the early growth stage of tomatoes, inoculation with *C. sorokiniana* significantly promoted tomato growth. However, as the plants matured, the growth-promoting effect of Bacillus combination gradually increased, becoming significantly superior to that of *C. sorokiniana* in the later growth stages. This advantage persisted until the end of the growth period. This indicates that different microorganisms exhibit varying mechanisms and effects at different stages of tomato growth. *C. sorokiniana* may confer growth advantages to tomatoes in the early stages through rapid colonization and nutrient competition. Research indicates that, beyond supplying inorganic nutrients, organic molecules present in *Chlorella vulgaris* culture supernatants and cell extracts can directly promote plant growth by stimulating root development (Wang et al., 2024). The sustained growth-promoting effects exhibited by Bacillus combination during the mid-to-late stages of crop development may be closely associated with their stable colonisation capacity within the rhizosphere environment. Through metabolic activities such as solubilizing phosphorus, mobilizing potassium, and fixing nitrogen, these bacteria convert insoluble soil nutrients into bioavailable forms. This process improves nutrient bioavailability and fertilizer efficiency, thereby ensuring a consistent nutrient supply for tomatoes during periods of rapid vegetative and reproductive growth. The sustained growth-promoting capacity of the *Bacillus* sp. SW14 strain—isolated from stress-tolerant environments—is supported by both genetic and phenotypic evidence: the identification of multiple growth-promoting genes (involved in nitrogen fixation, phosphorus solubilization, potassium mobilization, and IAA synthesis) within its genome, along with its significant improvement in key tomato growth indicators (Sadaiappan et al., 2025).

The results of this study indicate that treatment with *C. sorokiniana* and the Bacillus combination significantly increased the contents of chlorophyll a, chlorophyll b, total chlorophyll, and soluble sugars in tomato leaves. *C. sorokiniana* is rich in nutrients such as nitrogen and phosphorus, which can be utilized by plants to enhance chlorophyll synthesis. In plants, soluble sugars are multifunctional: they supply energy and carbon skeletons, regulate gene expression and signal transduction, maintain osmotic pressure, control photosynthesis and carbon allocation, influence leaf senescence and development, and contribute to microbial interactions and disease resistance (Sami et al., 2016). Research reports indicate that corn plants treated with *Chlorella vulgaris* also exhibit significantly increased chlorophyll a content and net photosynthetic rates, demonstrating its positive role in enhancing crop photosynthetic performance (Mieczyslaw and Romanowska-Duda, 2014). Inoculation with Bacillus subtilis RS10 and Bacillus pumilus SF-4 increased the total soluble sugar content in wheat by 28% and 25%, respectively (Iqbal et al., 2025). Additionally, this study revealed significantly reduced levels of soluble proteins and proline, suggesting that microbial inoculation may have induced the activation of systemic resistance in tomatoes. To counter such biotic stresses, plants must initiate defense responses. Consequently, the plants appear to have redistributed internal metabolic resources, shifting nitrogen and energy away from the synthesis of soluble proteins (including the osmotic regulator proline) and toward resistance-related physiological processes. As a result, the accumulation of proline was likely suppressed (Hare and Cress, 1997). As an osmoregulatory substance, proline plays a crucial role in crops by alleviating osmotic stress through maintaining cellular osmotic pressure (Dudziak et al., 2019). Therefore, a reduction in proline content may indicate that the treated plants are in a more stable physiological state and thus have a lower demand for osmotic regulation. When interacting with plant roots, *Chlorella* promotes phosphorus uptake by secreting organic acids but may indirectly inhibit nitrogen transport to the shoots, thereby affecting leaf protein synthesis (Win et al., 2018). Research has found that following the application of *Bacillus subtilis*, the proline content in cotton leaves first increases and then decreases. The proline content peaks during the boll formation stage, indicating that the osmotic regulation capacity of the leaves is strongest at this time. However, as the plants enter the boll opening stage, leaf senescence commences, leading to a reduction in proline synthesis (Bi et al., 2024).

To systematically evaluate the adaptability and tolerance of leaves following microbial treatment (resistance capacity of leaves), this study quantified key physiological and biochemical indicators across three dimensions: cell membrane stability, osmotic regulation and redox balance, and photosynthetic structural integrity. Specifically, the degree of lipid peroxidation in cell membranes was characterised by measuring malondialdehyde (MDA) content to assess membrane system stability; the accumulation of osmotic regulators such as proline and soluble sugars, alongside the activity of antioxidant enzymes including superoxide dismutase(SOD) and peroxidase (POD), were analysed to comprehensively reflect leaf osmotic regulation capacity and reactive oxygen species scavenging efficiency; simultaneously, chlorophyll content served as an indirect indicator of photosynthetic organelle structural and functional tolerance. Based on the systematic measurement and comprehensive analysis of these multidimensional indicators, the aim is to comprehensively quantify the physiological and biochemical adaptive capacity of leaves under microbial inoculation conditions. This study found that treatment with *C. sorokiniana* and Bacillus combination significantly enhanced the activity of antioxidant enzymes, including POD and SOD, in tomato leaves. This enhanced activity resulted in a lower MDA content compared to the CK, indicating a significant strengthening of the plant’s antioxidant defense capacity. While *C. sorokiniana* had no significant effect on CAT activity, Bacillus combination significantly enhanced it. POD, SOD, and CAT are crucial enzymes for plant adaptation and resistance to abiotic stress, as they are the key enzymes responsible for scavenging reactive oxygen species (ROS) and are collectively known as antioxidant enzymes. MDA is one of the primary products of membrane lipid peroxidation, reflecting the extent of membrane lipid peroxidation and indirectly measuring the degree of damage to the membrane system (Fang et al., 2021). Stress typically leads to the accumulation of large amounts of ROS within plants, including H_2_O_2_, OH^−^, and O_2−_. ROS are highly biologically active, which are capable of oxidizing biomolecules such as proteins, nucleic acids, carbohydrates, and lipids, thereby exerting toxic effects on plant cells (Gill and Tuteja, 2010; Hasanuzzaman et al., 2021). Enhanced antioxidant enzyme activity aids in scavenging ROS within plants, reducing MDA content, thereby mitigating oxidative damage. This enhanced resilience enables plants to maintain normal physiological metabolism under abiotic stresses like drought and low temperatures, which minimizes the inhibitory effects on growth. The mechanisms by which SOD and CAT alleviate oxidative stress have been extensively documented. At the cellular level, SOD catalyzes the dismutation of superoxide anion (O_2−_) into oxygen and hydrogen peroxide, thereby reducing oxidative stress levels. Subsequently, CAT further decomposes hydrogen peroxide into water and oxygen, effectively minimizing the potential damage posed by hydrogen peroxide to biomolecular structures within cells, such as proteins, lipids, and DNA. The dynamic equilibrium among the activities of SOD, ascorbate peroxidase (APX), and CAT in cells plays a crucial regulatory role in maintaining steady-state levels of superoxide radicals and hydrogen peroxide. This balancing mechanism, coupled with effective chelation of metal ions, is considered a vital physiological strategy for suppressing the generation of highly toxic hydroxyl radicals via metal-dependent Haber-Weiss or Fenton reactions (Singh, 2022). Multiple studies have demonstrated that microbial inoculation enhances plant stress resistance by activating their antioxidant defense systems. Research findings indicate that compared to the irrigation-only control group, the addition of *Chlorella vulgaris* significantly increased SOD activity by 49.25% (Wang et al., 2025). Under salt stress conditions, the composite bacterial agent Bacillus may enhance plant salt tolerance by inducing the activity of plant-specific stress-related enzymes such as SOD, POD, and CAT. Following inoculation with *Bacillus subtilis*, SOD content in heavily saline-alkali soils increased significantly by 237.46%, while MDA content decreased significantly by 47.30% (Xia et al., 2025). Potato plants treated with *Bacillus amyloliquefaciens* (FZB 24) and attacked by the wilt pathogen exhibited elevated SOD activity (Keerthana et al., 2018). The results of this study indicate that Bacillus combination exerted a significantly long-term growth-promoting effect on tomato plants compared to inoculation with *C. sorokiniana*. This effect was achieved by enhancing fundamental physiological indicators in leaves and increasing the activity of stress-related enzymes, thereby effectively advancing the plant’s healthy growth process.

### 4.2 Effects of *C. sorokiniana* and Bacillus combination on soil properties

This study evaluated the effects of *C. sorokiniana* and Bacillus combination on soil properties, concluding that the two treatments exerted distinct impacts. *C. sorokiniana* demonstrated advantages in enhancing SOC content and soil enzyme activity, while Bacillus combination had positive effects on soil nutrients (AN, AP, AK) and metal ion regulation. The efficacy of Bacillus combination aligns with previous findings that these inoculants, through rhizosphere colonization, provide sustained nutrient support during the mid-to-late stages of tomato growth via their capabilities in phosphorus solubilization, potassium mobilization, and nitrogen fixation.

After treatment with the *C. sorokiniana* and Bacillus combination inoculant, although the pH values differed significantly from CK, the actual change was minimal. This suggests that altered pH may not be the primary mechanism for plant growth promotion. Studies have shown that *Bacillus* inoculation significantly enhances rice seedling growth. However, in these studies, neither treatment with *Chlorella vulgaris* nor the Bacillus combination inoculant significantly affected soil physicochemical properties or pH (Nan et al., 2025). Soil EC decreased following both treatments, particularly in the *C. sorokiniana* group. This significant decrease is likely due to the uptake and assimilation of inorganic ions by *C. sorokiniana* during its growth and metabolism, which reduced the concentration of soluble ions in the soil solution. Consistent with these experimental findings, the *C. sorokiniana* treatment group significantly reduced the concentrations of Ca^2+^, Mg^2+^, and Na^+^ in the solution. In contrast, the Bacillus combination treatment group had no significant effect on Mg^2+^ concentration but significantly increased Ca^2+^ levels and markedly decreased Na^+^ content. These changes may reveal the specific effects of different treatments on metal element concentrations in soil, thereby reflecting their potential impact on soil chemistry and plant nutrition. For tomato growth, calcium and magnesium should be moderately elevated but guard against excessive concentrations that cause elemental imbalance, while sodium is best maintained at low levels. *Chlorella vulgaris*, as a photosynthetic autotrophic microalga, possesses cell surfaces rich in negatively charged groups. These enable the adsorption and uptake of cations from solutions through ion exchange mechanisms (Suresh Kumar et al., 2015). Research indicates that the absorption capacity of *Chlorella vulgaris* for Mg^2+^ and Ca^2+^ is closely related to its growth and metabolism. Mg^2+^ is a key element in chlorophyll synthesis and participates in the light reaction process of photosynthesis; Ca^2+^, meanwhile, plays a vital role in cellular signaling and regulating cell wall stability. Therefore, *C. sorokiniana* is expected to absorb Mg^2+^ and Ca^2+^ during growth, thereby reducing their concentrations in the environment. This mechanism is supported by experimental data. For instance, a study quantifying the adsorption and absorption of Mg^2+^ in a stirred photobioreactor found that biomass concentration positively correlated with Mg^2+^ removal efficiency, conforming to a reversible first-order kinetic model (Ben Amor-Ben Ayed et al., 2016). C*. sorokiniana* may reduce soil Na^+^ concentration through ion exchange and competitive absorption. Its secretions and residues improve soil structure and immobilize Na^+^, while its growth alters soil pH, affecting Na^+^ dissolution and precipitation, ultimately lowering soil electrical conductivity—a finding consistent with our research. Previous studies indicate that during growth, *Chlorella vulgaris* exhibits a preference for absorbing mineral nutrients such as K^+^, Ca^2+^, and Mg^2+^ (Ismail et al., 2011). This may reduce Na^+^ uptake and indirectly decrease the activity and availability of sodium ions in the soil. The effect of the Bacillus combination inoculant on increasing Ca^2+^ ion content may be related to its metabolites. During metabolism, the Bacillus combination inoculant may secrete substances such as organic acids or extracellular polysaccharides, which can form complexes with Ca^2+^, thereby altering Ca^2+^ solubility. This complexation may increase the free Ca^2+^ concentration in solution, leading to elevated detected Ca^2+^ levels. Studies indicate that when nitrogen-fixing bacteria containing plant growth-promoting rhizobacteria (PGPR) are applied to soil, plant K^+^, Ca^2+^, and Mg^2+^ content significantly increases (Matse et al., 2020).

In this experiment, compared with CK, both treatments resulted in increased soil organic matter content. Furthermore, the *C. sorokiniana* treatment group exhibited a significantly greater increase in organic matter content than the Bacillus combination treatment group. This finding is consistent with previous research results (Alobwede et al., 2019). Although the inoculation of *C. sorokiniana*, which is rich in amino acids and oligopeptides, was expected to promote microbial proliferation and increase soil organic matter, the observed decreases in soil AN, AP, and AK following inoculation suggest a more complex interaction. The increase in organic matter might be initial, while the decline in available nutrients could be due to intense microbial immobilization or rapid uptake by plants. Conversely, Bacillus combination treatment group significantly increased these three nutrients, indicating its role in promoting the release of AN, AP, and AK elements, enhancing soil fertility, and thereby facilitating the restoration of degraded soil environments. Research findings indicate that untreated tomato seedlings exhibited significantly lower accumulation levels of total N, P, and K compared to the other four treatments. Compared to seedlings receiving only nutrients, tomato seedlings inoculated with *Bacillus velezensis* alone exhibited significantly higher total potassium accumulation and equivalent accumulation of total N, P (Peng et al., 2025). Additionally, the inoculation of *Bacillus subtilis* significantly enhances tomato plants’ phosphorus uptake and growth by increasing the availability of phosphorus in the soil (Shao et al., 2025). Following the application of *C. sorokiniana*, the levels of alkali-hydrolyzable nitrogen, readily available potassium, and readily available phosphorus all decreased. This may be attributed to *C. sorokiniana* absorbing substantial amounts of nutrients such as nitrogen, phosphorus, and potassium during its growth, thereby reducing the content of these readily available nutrients in the soil (Asaad and Amer, 2024). Research has found that potassium concentrations decrease in soil amended with *C. sorokiniana* leachate (Garbowski, 2024).

In our study, the inoculation of *C. sorokiniana* significantly increased the activity of urease and protease in the soil but decreased invertase activity. In contrast, the inoculation of the Bacillus combination inoculant significantly increased the activities of invertase, urease, and protease in the soil. Notably, the activities of urease and protease were significantly higher in soils inoculated with *C. sorokiniana* than in those inoculated with the Bacillus combination. Furthermore, neither treatment significantly affected catalase activity. This differential response reflects distinct regulatory mechanisms of the two biopesticides on soil microbial communities and nutrient cycling processes. Soil enzymes are a vital component of the soil system, participating in most biochemical reactions within the soil. Soil enzyme activity reflects the rate and intensity of these biochemical reactions and contributes to the stability of the soil system. Soil enzyme activity is one of the key indicators of soil quality (Raiesi and Salek-Gilani, 2018). Soil urease plays a vital role in soil nitrogen cycling by catalyzing the hydrolysis of organic nitrogen into plant-available ammonium, thereby improving nitrogen bioavailability (Liu et al., 2022). Soil organic nitrogen primarily exists as proteins, and its bioavailability depends on the depolymerization of these proteins into peptides and free amino acids. Consequently, protease activity is the key rate-limiting factor governing organic nitrogen mineralization (Caballero et al., 2020). Soil invertase catalyzes the hydrolysis of sucrose into glucose and fructose, providing readily available carbon sources for soil microorganisms. As a key enzyme driving soil carbon cycling and energy flow, its activity is widely used as a core biological indicator for measuring soil fertility (Gu et al., 2009). Based on the experimental results, we can conclude that the specific effect of *C. sorokiniana* on soil enzyme activity primarily manifests as its strong stimulation of nitrogen cycle-related enzymes. In contrast, the broad-spectrum promotion of soil enzyme activity by the Bacillus combination is more comprehensive.

### 4.3 *C. sorokiniana* and Bacillus combination regulate the structure and function of the rhizosphere soil microbial community

It is well known that the inoculation of beneficial microorganisms in agriculture impacts soil microbial diversity, which serves as one of the key indicators for assessing soil health and quality (Ju et al., 2021). In-depth investigation of the dynamic changes in bacterial communities during plant growth in soil is crucial for comprehensively understanding growth-promoting mechanisms. High-throughput sequencing analysis of samples can reveal species composition (Du et al., 2025). The Shannon index for Bacillus combination inoculation was higher than that for *C. sorokiniana* inoculation, indicating greater microbial community diversity; however, the Chao and Ace indices were lower than those for *C. sorokiniana* inoculation, suggesting lower species richness with Bacillus combination inoculation. The Simpson index was lower than that of *C. sorokiniana* inoculation, further indicating more even species distribution after Bacillus combination inoculation. This may be because the soil environment became more stable after Bacillus combination inoculation, facilitating uniform microbial distribution. Although species numbers were lower, ecological functions were more stable. In contrast, *C. sorokiniana* inoculation resulted in high species richness but uneven distribution, leading to relatively weaker ecological function stability.

Following inoculation with *C. sorokiniana* and the Bacillus combination inoculant, the dominant phylum composition of the soil microbial community was broadly similar, although differences existed in the relative abundance of individual phyla. Inoculation with both treatments increased the abundance of Planctomycetes. This phylum participates in soil nitrogen cycling and aggregate formation, exhibiting unique anaerobic, autotrophic metabolism, including the ability to oxidize ammonium nitrogen (Fuerst and Sagulenko, 2011). Following inoculation with *C. sorokiniana*, the abundance of Pseudomonas, Bacillota, and Nitrospirota increased. The Pseudomonas phylum plays a crucial role in soil ecosystems. Numerous strains within the Pseudomonas phylum possess the ability to inhibit the growth and reproduction of plant pathogens in soil (Bernal et al., 2017). They suppress pathogens by producing antibiotics and hydrolytic enzymes to combat pathogens like *Fusarium graminearum* (Arseneault and Filion, 2016), while simultaneously promoting plant growth by secreting auxins and phytohormones that enhance root development and nutrient uptake (Vessey, 2003; Lugtenberg and Kamilova, 2009). *Bacillus* species exhibit rich genetic and metabolic diversity, playing a pivotal role in plant health and soil ecological balance through multiple beneficial traits. These include promoting nutrient cycling, enhancing plant growth, strengthening plant stress resistance, and defending against pathogens and abiotic stresses within soil ecosystems (Saxena et al., 2020). Nitrifying spirillum plays a crucial role in the nitrification process. Its genome possesses the molecular mechanisms for ammonia oxidation and nitrite oxidation, enabling it to perform a key function in the nitrogen cycle (Vijayan et al., 2021). Additionally, unlike after inoculation with *C. sorokiniana*, inoculation with the Bacillus combination inoculant significantly increased the abundance of Gemmatimonadota, Bacteroidota, Chloroflexota, Actinomycetota, and Cyanobacteriota. Gemmatimonadota exhibits widespread distribution and diverse metabolic capabilities, playing a crucial role in biogeochemical cycles. Its abundance correlates positively with certain soil nutrients and demonstrates environmental preferences and functional adaptability (Du et al., 2024). Bacteroidetes efficiently degrade cellulose and hemicellulose, promoting organic matter transformation and humus formation (Gavande et al., 2021). Chloroflexi are oligotrophic bacteria that degrade cellulose and can adapt to nutrient-poor environments (Hu et al., 2024). Cyanobacteriota can produce extracellular polysaccharides that promote soil particle aggregation, increase soil organic matter content, and improve soil physical structure. They also generate oxygen and organic matter through photosynthesis, providing nutrients for plants, while secreting plant hormones to stimulate plant growth (Nawaz et al., 2024; Singh et al., 2016). Consistent with the findings of this study, inoculation with the Bacillus combination inoculant exerts sustained positive effects on tomato plant growth by selectively regulating soil microbial community structure. Tomato plants exhibited enhanced growth vigor and improved stress tolerance throughout extended growth periods, particularly maintaining favorable physiological states during environmental fluctuations. Soluble sugar content within plants also significantly increased, indicating that inoculation not only optimized the rhizosphere microenvironment but also systematically enhanced plant carbon assimilation and stress adaptation capabilities.

Through an in-depth analysis of the relationship between quantitative environmental factors and rhizosphere soil bacterial community structure, we found that SOC, protease, and POD play key roles in shaping the bacterial community structure of *C. sorokiniana* rhizosphere soil. Regarding the construction of rhizosphere bacterial communities by the Bacillus combination, soluble sugar, CAT, Ca2+, alkaline nitrogen, and available potassium are the primary driving factors. This indicates that the formation of *C. sorokiniana* rhizosphere bacterial communities is closely related to soil SOC content and microbial protease and POD enzyme activities. These factors collectively shape the unique bacterial community structure in this plant’s rhizosphere, thereby influencing plant growth, health, and the acquisition and utilization of soil nutrients. Conversely, the formation of the Bacillus combination bacterial community in rhizosphere soil is influenced by the combined effects of multiple soil nutrient elements and microbial activities. These factors interact to jointly determine the structural and functional characteristics of this rhizosphere bacterial community.

EC is a key factor influencing bacterial diversity and community structure in greenhouse soils, highlighting the regulatory role of electrical conductivity on microbial communities under specific soil conditions (Kim et al., 2016). Relevant studies have investigated the impact of hydrological factors on bacterial diversity and community composition in desert river sediments. Findings indicate a strong correlation between electrical conductivity and bacterial diversity, suggesting that electrical conductivity exerts a significant influence on microbial communities (Zeglin et al., 2011). This conclusion is supported by our correlation analysis between soil microbial diversity and environmental factors, which revealed a significant positive correlation between the phylum Patescibacteria and EC. Following inoculation with either *C. sorokiniana* or the Bacillus combination inoculant, the abundance of Patescibacteria decreased. Notably, inoculation with *C. sorokiniana* resulted in the most rapid decline, which was associated with a significant reduction in EC values. Patescibacteria are known to depend on hosts, such as actinomycetes, for growth, potentially utilizing host-provided nutrients or thriving in high ionic strength environments. Thus, elevated EC values may enhance host actinomycete activity, indirectly supporting Patescibacteria growth (Wang et al., 2023). Therefore, we can infer that the abundance of the Patescibacteria phylum in the patella is directly related to EC.

Overall, compared to inoculation with *C. sorokiniana*, inoculation with the Bacillus combination inoculant significantly enhances the abundance of dominant microbial species. This, in turn, strengthens the soil’s ecological functions across multiple domains, including nitrogen and carbon cycling, organic matter decomposition and transformation, humus formation, and maintenance of microbial community stability. These improvements contribute to enhanced soil environmental quality and overall soil health.

### 4.4 Systemic co-variation between plants and soil: Integrative evidence from rhizosphere colonization assessment and yield prediction

The congruent systemic alterations in plants and soil provide robust evidence to assess microbial rhizosphere colonization and predict yields. Firstly, the sustained improvement in plant growth and physiological indicators (consistent increases in plant height and stem thickness, alongside significant elevations in chlorophyll content and stress-resistant enzyme activity) aligns closely with the biological expectations of successful microbial colonization (Wang et al., 2025; Brilli et al., 2019; Hsu and Micallef, 2017, Gayathri et al., 2025). The marked differences compared to uninoculated controls rule out environmental chance factors, confirming these changes are driven by the introduced active microorganisms. Had microbial colonisation been ineffective, their growth-promoting effects would have exhibited transient, diminishing characteristics rather than the persistent and systemic pattern observed in this study. Consequently, these continuously enhanced plant responses may be regarded as indirect yet compelling correlative evidence that microorganisms have established stable niches within the rhizosphere and exert sustained functional effects. Secondly, the dynamic alterations in rhizosphere soil properties exhibited a pronounced temporal correlation with improvements in plant growth indicators. The inoculation treatment significantly modified the structure of the rhizosphere microbial community (alpha and beta diversity) while concurrently enhancing soil available nutrients (nitrogen, phosphorus, potassium) and key enzyme activities (urease, protease). Colonizing microorganisms, through their active metabolism, can reshape the rhizosphere microenvironment, leading to this suite of alterations in soil parameters (Yang et al., 2026). The temporal coupling between the dynamic soil improvement process and enhanced plant growth indicators further reinforces the causal chain: microbial colonisation → soil function optimisation → enhanced plant nutrition and resistance.

In discussions concerning yield, we have further clarified the logical relationship between measured physiological parameters and yield potential. Existing literature indicates that the synergistic enhancement of indicators such as plant vigour, photosynthetic capacity, stress tolerance, and soil fertility is typically positively correlated with crop yield (He et al., 2025; Luo et al., 2025). Previous studies have confirmed that chlorophyll content is one of the key physiological drivers influencing crop yield, exhibiting a stable positive correlation with final economic yield (Hu et al., 2025; Wang et al., 2019). In this experiment, the inoculation treatment significantly and persistently enhanced tomato vegetative growth, while also increasing chlorophyll content and leaf resistance enzyme activity. These findings indicate that the plants achieved a healthier and more vigorous physiological state. This aligns with the established conclusion that high photosynthetic capacity and robust vegetative growth are prerequisites for high yield (Chen et al., 2024; Zhang et al., 2025). Therefore, the systemic physiological improvements induced by inoculation—enhanced photosynthesis and promoted biomass accumulation, which supply more abundant assimilates for fruit development—provide a solid logical basis for predicting eventual yield increases (Liu et al., 2022; Appolloni et al., 2021). Consequently, the markedly enhanced indicators induced by inoculation and systematically monitored in this study may be regarded as reliable leading indicators for predicting high-yield potential.

In summary, the systemic synergistic changes occurring at the rhizosphere level—encompassing morphological, physiological, and microbial community aspects—constitute an integrated process of plant-soil interaction. This process not only reflects the successful establishment of microbial communities but also drives efficient crop growth and yield formation. By monitoring these synergistic changes across multiple dimensions, we can more scientifically assess rhizosphere health, predict agricultural productivity, and provide a core theoretical foundation for designing efficient green agricultural technologies.

### 4.5 Evidence for sustainability: the long-term significance of microbial inoculation for soil health and agricultural productivity

This study reveals that the application of Bacillus consortia and *C. sorokiniana* exerts differential effects on the tomato rhizosphere microenvironment, with these alterations being closely linked to soil health and agricultural sustainability. Although long-term field validation and multi-seasonal trials are essential to confirm their application stability, this research uncovers mechanisms of significant sustainability implications at the biological process level.

First, enhanced microbial diversity and strengthened ecosystem stability. The inoculation treatment significantly increased the Shannon diversity index of rhizosphere bacterial communities. As a key indicator of soil ecosystem stability, increased microbial diversity signifies enhanced functional redundancy and disturbance resilience. This not only mitigates microbial community degradation caused by continuous cropping or environmental stress but also provides a biological foundation for sustaining critical ecological functions such as soil nutrient cycling and disease suppression over the long term.

Second, differential promotion of key biochemical processes. The two inoculation treatments exhibited distinct functional characteristics: The *C. sorokiniana* treatment significantly increased total soil organic carbon content and nitrogen cycle-related enzyme activity, but reduced available nitrogen, phosphorus, and potassium levels. This may relate to its carbon-fixing metabolic priority and functional role in promoting carbon-nitrogen conversion. Conversely, the Bacillus combination comprehensively elevated soil available nitrogen, phosphorus, and potassium levels along with nutrient availability, demonstrating stronger nutrient mobilization capacity. This functional complementarity offers potential for constructing synergistic microbial systems.

Third, a comprehensive analysis of multiple datasets indicates that the application of *C. sorokiniana* demonstrates potential for alleviating soil salinity stress and improving soil conditions. Compared to the control group, soil pH exhibited a decreasing trend within the alkaline range following inoculation, suggesting a possible mitigating effect on alkalinity stress and enhanced hydrogen ion activity. This may contribute to improved nutrient availability and stimulated microbial activity. Concurrently, reductions in soil electrical conductivity and sodium ion content suggest a possible decrease in salt concentration. This may help alleviate plant water uptake inhibition and ion toxicity issues arising from excessively low soil solution osmotic potential. Moreover, the concurrent reduction in sodium, magnesium, and calcium ion concentrations further suggests that *C. sorokiniana* may possess a composite function of absorbing or promoting the leaching of salt-separating ions. This could collectively reduce soil salinity loads and mitigate ion stress intensity. Collectively, these findings demonstrate *C. sorokiniana*’s potential application in saline-alkali land remediation. They provide a reference for further exploration of its use as a green bio-amendment in sustainable agriculture, holding positive implications for ecological restoration of saline-alkali soils and research into sustainable arable land utilisation.

Collectively, the “biological sustainability” responses revealed in this study—encompassing increased microbial diversity, differentiated activation of key enzyme systems, and complementary optimization of soil nutrient pool functions—constitute crucial scientific evidence for assessing the sustainability of microbial fertilizers or biostimulants. These changes indicate that inoculating microorganisms not only promotes plant growth in the short term but also potentially lays the foundation for the long-term health and productivity of agricultural ecosystems by improving soil biological quality. Introducing functionally distinct microbial communities into tomato cultivation represents an effective strategy not only for mitigating environmental stresses and reducing synthetic inputs, but also a crucial pathway towards ecologically intensive agriculture. This study offers theoretical and technical support for achieving sustainable and stable crop production through ecological mechanisms, especially in the face of global climate change (Benaissa et al., 2025; Xu et al., 2026).

### 4.6 Research limitations and future prospects

This study employed multi-indicator collaborative observation system to systematically compare and analyse the differences between inoculating *C. sorokiniana* and Bacillus combination on tomato plants across four dimensions: the persistence of growth-promoting effects, improvements in rhizosphere soil physicochemical properties, systemic resistance induction capacity, and overall regulation of the rhizosphere microenvironment. This provides crucial empirical evidence for elucidating the growth-promoting mechanisms of different microbial groups. Nevertheless, this study retains the following significant limitations: First, it did not include detailed soil property data, which are key covariates influencing microbial inoculation effects. This limitation may weaken the generalizability of findings across different agricultural systems or ecological regions. Second, regarding microbial colonisation verification, molecular biological techniques such as fluorescent labelling and quantitative PCR were not employed to directly observe and quantitatively analyse the dynamic colonisation process of inoculated strains within the rhizosphere. Finally, the agronomic assessment failed to cover the entire growth cycle of tomatoes, resulting in the absence of a systematic evaluation of final yield components and fruit quality traits. This limitation restricts the accurate assessment of the practical application potential. To advance research in this field, subsequent studies should integrate modern microbial tracing and functional quantification techniques to directly elucidate the colonisation dynamics, spatial distribution, and metabolic activity of inoculated microorganisms within the rhizosphere. Concurrently, field trials spanning multiple seasons, diverse soil types, and the entire crop cycle must be designed to comprehensively evaluate the long-term impacts on crop yield, quality, and soil health. These efforts should progressively extend to diverse cropping systems and ecological environments to enhance the scientific rigour, robustness, and practical applicability of research conclusions.

## 5 Conclusion

This study compared the effects of Bacillus combination microbial inoculant and *C. sorokiniana* on tomato plant growth and soil-related indicators. Results indicate that Bacillus combination inoculant exhibits a significantly longer-lasting growth-promoting effect on tomato plants compared to *C. sorokiniana* inoculation. This effect primarily occurs by enhancing fundamental physiological indicators in leaves and increasing the activity of stress-related enzymes, thereby effectively advancing the plant’s healthy growth process. Regarding soil, *C. sorokiniana* demonstrated advantages in increasing SOC content and nitrogen cycle-related enzymes. In contrast, the Bacillus combination inoculant exerted positive effects on soil nutrient accumulation (e.g., AN, AP, AK) and metal ion regulation, while also exerting more favorable regulatory effects on rhizosphere soil microorganisms. Additionally, we observed a potential direct correlation between Patescibacteria abundance and reduced EC. These findings contribute to elucidating the underlying mechanisms of functional differences among microbial inoculants. Such understanding is crucial for exploring how microbial inoculants promote crop growth and for determining scientifically sound methods to optimize their efficacy. However, precisely regulating interactions between exogenous microbial inoculants and rhizosphere microbial communities remains an unresolved challenge. Future research may employ metagenomic analysis and genetic validation to delve deeper into the potential mechanisms underlying interactions between microbial inoculants and rhizosphere microbial communities, as well as their pathways of influence on plant health. Through empirical analysis, this study reveals significant variations in plant growth promotion, soil properties, microbial diversity, and network complexity among algal-bacterial microbial preparations. The findings suggest that these preparations can be rationally applied according to specific scenarios, serving as a key strategy for enhancing plant growth and advancing sustainable agricultural development. This study also evaluated the regulatory effects of *C. sorokiniana* on tomato plant growth and preliminarily explored its potential mechanisms of action, aiming to provide theoretical underpinnings and practical guidance for developing highly efficient, novel microalgae-derived biofertilisers.

## Data Availability

The original contributions presented in the study are publicly available. This data can be found here: NCBI, BioProject ID: PRJNA1453515.
